# Development and psychometric testing of a scale to measure effective rural emergency transfer (RET)

**DOI:** 10.1186/s12873-024-01046-2

**Published:** 2024-07-29

**Authors:** Tebogo T. Mamalelala, William L. Holzemer, Esther S. Seloilwe, Emilia Iwu

**Affiliations:** 1 Faculty of Health Sciences, School of Nursing, University of Botswana, Gaborone, Botswana; 2 School of Nursing, The State University of New Jersey, Rutgers, Newark USA; 3 Faculty of Health Sciences, School of Nursing, Private bag 00712, Gaborone, Botswana

**Keywords:** Rural health facilities, Scale development, Categorical principal components analysis

## Abstract

**Background:**

The process of transferring patients from small rural primary care facilities to referral facilities impacts the quality of care and effectiveness of the referral healthcare system. The study aimed to develop and evaluate the psychometric properties of a scale measuring requirements for effective rural emergency transfer.

**Methods:**

An exploratory sequential design was utilized to develop a scale designed to measure requirements for effective emergency transport. Phase one included a qualitative, interview study with 26 nursing transport providers. These transcripts were coded, and items developed for the proposed scale. Phase two included a content validity review by these 16 transport providers of the domains and items developed. Phase three included development and evaluation of psychometric properties of a scale designed to measure requirements for effective emergency transport. This scale was then tested initially with 84 items and later reduced to a final set of 58 items after completion by 302 transport nurses. The final scale demonstrated three factors (technology & tools; knowledge & skills; and organization). Each factor and the total score reported excellent scale reliability.

**Results:**

The initial item pool consisted of 84 items, generated, and synthesized from an extensive literature review and the qualitative descriptive study exploring nurses’ experiences in rural emergency patient transportation. A two-round modified Delphi method with experts generated a scale consisting of 58 items. A cross-sectional study design was used with 302 nurses in rural clinics and health in four rural health districts. A categorical principal components analysis identified three components explaining 63.35% of the total variance. The three factors, technology, tools, personal knowledge and skills, and organization, accounted for 27.32%, 18.15 and 17.88% of the total variance, respectively. The reliability of the three factors, as determined by the Categorical Principal Component Analysis (CATPCA)’s default calculation of the Cronbach Alpha, was 0.960, 0.946, and 0.956, respectively. The RET Cronbach alpha was 0.980.

**Conclusions:**

The study offers a three-factor scale to measure the effectiveness of emergency patient transport in rural facilities to better understand and improve care during emergency patient transport.

**Supplementary Information:**

The online version contains supplementary material available at 10.1186/s12873-024-01046-2.

Referral is a process in which health professionals with insufficient resources at one level of the health system seeks the aid of a better or differently resourced facility at the same or higher level to manage a clinical condition [1]. The referral system in primary health care promotes equitable access to secondary and tertiary health care, and its implementation influences the efficacy and quality of health care delivery in rural areas [2]. Most countries’ healthcare systems are designed to encourage patients to seek treatment at the primary health level before progressing to a higher level of care if the care required exceeds that of a primary care level facility [3]. Transferring patients from small rural primary care facilities to referral facilities evaluates the effectiveness of the referral healthcare system [3, 4]. An effective referral system ensures that all levels of health care collaborate to provide individuals with the best possible care [3].

Botswana’s government provides free healthcare to its people. However, foreign nationals must pay a fee. Healthcare services in rural areas of Botswana are provided through an extensive national network of health facilities consisting of a referral system. It starts with primary care including 931 mobile stops, 351 health posts and 311 clinics and progresses to 17 primary hospitals, seven public district and three tertiary level hospitals [5]. Seven hundred thirty thousand five hundred ninety-one individuals (27.78%) live in the country’s rural areas with a substantially lower population density of 4.1 persons per km^2^ [6]. Generally, cities and towns have the highest population densities compared to predominantly rural districts. Hospitals are more concentrated and geographically skewed toward the north and southeast parts of the country, and there are fewer primary and district hospitals in sparsely populated large geographic areas, impacting timely access to healthcare services [7]. The public and private sectors operate Emergency Medical Services (EMS); however, it is still in its infancy and only covers the cities and significant villages [8]. Registered nurses and midwives in rural Botswana, lacking designated patient transport roles, escort patients between health facilities without specific training [9].

The rural emergency transport process has been associated with system failures and safety hazards such as extended distances and inadequate infrastructure, feelings of isolation, a lack of technology and tools, weather challenges, and a lack of system coordination and manager support [4, 10]. Moreover, rural nurses are responsible for decision-making, critical thinking, prioritization of care, and transfer. Nurses usually serve as the health care provider during transport and must have mastered non-technical skills like flexibility and adaptation to function effectively in a demanding remote environment. While clinical knowledge, experience, and skills have a significant impact on emergency care personnel’s professional capacity to make clinical judgments [11, 12], most of these skills are not formally taught in nursing school but are acquired on the job.

Despite several studies indicating the challenges of patient transport in sparsely populated rural areas [4, 12, 13], to the best of our knowledge, no instrument exists for measuring effectiveness of rural emergency patient transport. Standardizing and validating procedures is essential to measure and evaluate the effectiveness of emergency patient transport during rural emergency transport. This will help improve emergency rural transport and clinical practices. The study aimed to develop and evaluate the psychometric properties of a scale measuring requirements for effective rural emergency transfer.

## Purpose

The aim of the present study was to develop and evaluate the psychometric properties of a scale measuring effective rural emergency transfer. The specific aims of this project include:

1. Investigate the opinions of experts in emergency care fields and obtain consensus on the requirements of nurses to undertake safe emergency patient transport in Botswana.
2. Identify the barriers, gaps, and challenges in managing acute and emergency medical conditions.
3. Identify training needs based on the current educational demands of transport nurses proposed by the experts through a survey.
4. Examine the psychometric properties of a scale measuring emergency patient transport safety by nurses in rural health facilities in Botswana.

## Methods

### Research design

**Fig. 1:**
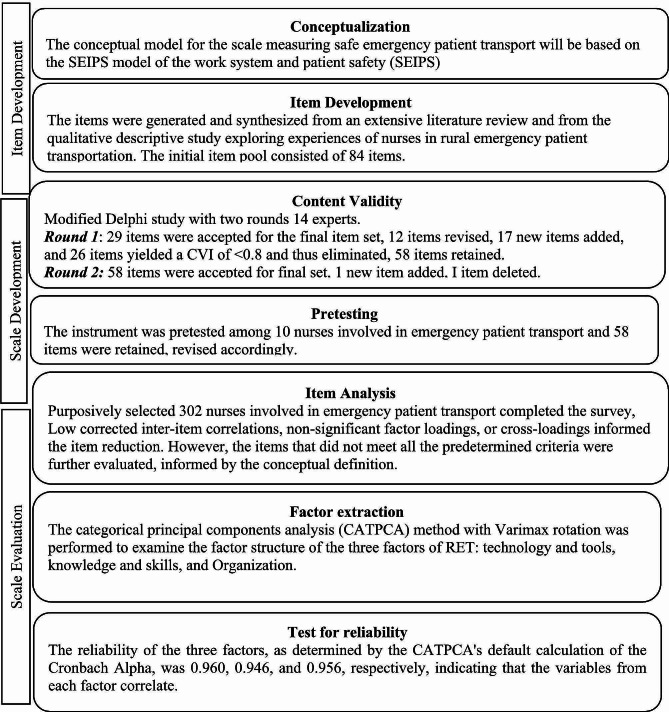
An overview of scale development and psychometric testing

Three methodologies were utilized to develop a scale designed to measure effectiveness of emergency patient transport. Three methodologies were utilized to develop a scale designed to measure requirements for effective emergency transport [14]. developed a framework to guide the three stages of scale development: item development, scale development, and scale evaluation (see Fig. 1). Phase one was the qualitative descriptive study exploring nurses’ experiences while undertaking emergency transportation of patients from rural health posts and clinics to generate the items. Phase two built upon the qualitative descriptive study, involved content validity using two round modified Delphi study with 16 experts whereas phase three was scale evaluation including pre-testing the items, sampling and survey administration, item reduction, and extraction of factors [14] using a cross-sectional study design among a sample of 302 nurses in rural clinics and health posts undertaking emergency patient transport in four health districts.

### Data collection

#### Study setting

Data for phase one and phase three were collected in four selected remote rural health districts, including Boteti, Kweneng West, Kgalagadi North, and Okavango. The four districts have five hospitals, 26 clinics and 60 health posts.

#### Phase one: identification of domain and item generation

The items were generated and synthesized from the qualitative descriptive study [4] and extensive literature review. The conceptual model for the scale measuring safe emergency patient transport was based on the SEIPS model of the work system and patient safety (SEIPS). The model is practice-driven and asserts that work systems are multidimensional and comprise five distinct dimensions: person, technology and tools, tasks, organization, and physical environment [15]. (**see** Table 1**).**

**Table 1:** Dimensions and their definitions

Dimension	Definitions
**Knowledge and Skills**	The ability to apply knowledge to specific situations by nurses working in rural clinics and health posts undertaking emergency patient transport.
**Technology and Tools**	Objects used to assist persons in performing tasks. Tool and technology factors in the SEIPS model can be characteristics such as usability, accessibility availability, and portability.
**Organization**	The structure that provides and coordinates time, space, resources, and activities.

#### Phase two: content validity

Phase two utilized modified Delphi utilizing 16 respondents, to ascertain consensus on the components of effective emergency patient transport from rural health facilities in Botswana by examining the viewpoints of experts in the field of emergency care. The data was collected by the first author (TM). The experts were presented with a list of items developed from round one, a qualitative descriptive study. Experts were asked to rate their agreement on whether to include each item on a five-point Likert scale strongly agree/ agree/ disagree/strongly disagree). Additionally, they were asked to comment on the wording and clarity and to make suggestions for additional criteria they consider to be included. Furthermore, experts were asked to indicate any overlap between items and suggest whether some items might be combined.

##### Sampling

 Participants were selected from various specialists in emergency medicine, trauma surgery, trauma, emergency nursing, nurses, and EMTs who transport patients, including academic and clinical knowledge, to achieve heterogeneity within the discipline [16, 17]. The contact information for specialist providers (nurses, paramedics, and emergency medicine) in the country will be requested from registration bodies such as the Nursing and Midwifery Council of Botswana (NMCB) and the Botswana Health professional council. To generate a snowball effect, experts will also be requested to extend the request to potential participants in their professional networks that might meet the inclusion criteria.

##### Data Analysis

 Descriptive statistics and content analysis were used to analyze data from round one and two. Frequencies for the responses were calculated for the importance of each item across all participants. Consensus for the inclusion were considered if an item is rated “strongly agree” or “agree” by at least 80% of the experts. Data were analyzed using IBM SPSS version 29 using descriptive analysis (frequencies). Free-text comments were analyzed using content analysis and used to refine and expand the set of criteria. Items that received less than 80% agreement or underwent significant modification were relisted in the following Delphi round, along with succinct justifications for or against inclusion Keeney, Hasson [18]. The list for the following Delphi round were updated to include suggested missing items.

##### Phase three: scale evaluation (main study)

This Phase of the study evaluated the psychometric properties of items in the newly developed scale including pre-testing the items, sampling and survey administration, item reduction, and extraction of factors [14] using a cross-sectional study design among a sample of 302 nurses in rural clinics and health posts undertaking emergency patient transport in four health districts. The development of a reliable and valid scale is important to empirical research and leads to more accurate research findings [19].

Data were collected for seven weeks, from July to end of August 2023. The researcher followed the nurses at their work settings, the rural health facilities, invited the nurses to participate and requested the participants to complete the questionnaire before the researcher’s return, and the researcher collected all the completed questionnaire immediately after completion.

##### Sampling

 All eligible nurses on duty during the study period were invited to participate. The scale to measure effective emergency patient transport in resource limited settings was a paper based self-reported paper-based survey including 58 items. The Scale used a bipolar Likert rating scale with descriptors measuring five levels of agreement (strongly disagree to strongly agree). Participants were instructed to reflect on their own patient transfer experience in answering each statement (item), with higher scores indicating higher agreement on Effective Rural Emergency Transport (RET).

##### Data analysis

 The suitability of Principal Component Analysis (PCA) was assessed prior to analysis. An exploratory factor analysis was run on the 30-item scale, the scale met the assumptions for EFA with the KMO index value of 0.946 and a significant Bartlett’s test of sphericity (*p* < 0.001) indicating that the data was likely factorizable.

##### Item Reduction

 The selection of items was an iterative process performed by the researcher. Low corrected inter-item correlations, non-significant factor loadings, or cross-loadings informed the item reduction. However, the items that did not meet all the predetermined criteria were further evaluated, informed by the conceptual definition. The Categorical Principal Component Analysis (CATPCA) presents some items with cross-loading, possibly due to the non-linear relationships between the variables that this technique enables us to consider. All cross-loadings of the CATPCA results are greater than 0.3. In all cases of cross-loadings, the best approach would be to delete these items and perform a new analysis, and 30 items retained.

##### Factor Extraction of the RET Scale

 The Categorical Principal Components Analysis developed by the Faculty of Social and Behavioral Sciences, Leiden University, The Netherlands, was implemented in SPSS, version 29.0, which is an implementation of the optimal scaling approach to Nonlinear Principal Components Analysis (NLPCA). Categorical Principal Component Analysis (CATPCA) is appropriate for nominal or ordinal variables. It is beneficial for ordinal data such as the Likert Scale [20, 21], which was helpful in this study since the data scoring level for the RET Scale was ordinal.

The Dimension Reduction option offers a more interactive method called the CATPCA. The relationship between the variables was optimized using the Variable Principal approach. Kaiser Normalization was used with Varimax as the rotation method. The Optimal Scaling procedure depends on the number of dimensions; hence, there was no automatic criterion for the extraction factor. The variable map, a graphical representation of the factorial solution, various numbers of dimensions using the criteria of an eigenvalue greater than 1, and an interpretability factor were used to determine the ideal number of dimensions to extract.

## Results

### Phase 1

The items were generated a from the qualitative descriptive study involving 26 nursing transport providers. These transcripts were coded, and 84 items developed for the proposed scale [4]. The interviews explored the range of skills and knowledge nurses use on the job, the complex needs of this demographic, the resources they need, and the problems they encounter in certain situations. Dimensions and their definitions are provided in Table 1.

### Phase 2

Data were collected between May and June 2023. Sixteen experts were invited to participate in both rounds. Fourteen experts participated in round one (88% response rate), and 15 respondents participated in round two (93.8% response rate). Sex distribution was consistent across all two rounds, with a higher percentage of males than females (> 64%). Most of the experts specialized in emergency medicine (43%, *n* = 6) compared to paramedics (28.5%, *n* = 4) and emergency nursing (28.5%, *n* = 4). More than half of the experts worked in the clinical setting and had a master’s degree. **See** Table 2. The initial item pool consisted of 84 items according to the six domains, as follows: 18 transporter items, 24 tasks-related items, 15 tools and technologies items, 14 organizational items, 7 environment related items, and 6 outcome related items.

**Table 2:** *Descriptive data from participants in the qualitative interviews (round 1;* *N* *= 14)) and validity assessments (round2:* *N* *= 15)*

		Round 1		Round 2	
		n	%	n	%
Number of respondents invited		16	100%	16	100%
Response rate		14	88%	15	93.8%
Sex	Female	5	35.7%	5	33.3%
	Male	9	64.3%	10	66.7%
Age	31–40	1	7%	2	13.3%
	41–50	9	64.3%	9	60.0%
	51–60	4	28.6%	4	26.7%
Professional Designation	Emergency Nurse	4	28.5%	5	33.3%
	Paramedic	4	28.5%	4	26.7%
	Emergency Physician	6	43%	6	42.9%
Primary Site of Work	Academic setting	3	21%	3	20.0%
	Clinical setting	8	57%	9	60.0%
	Both academic and clinical	3	21%	3	20.0%
Highest level academic of qualification	Bachelor’s degree	6	42.9%	6	40.0%
	Master’s degree	8	57.1%	9	60.0%

***Round one.*** Eighty-four items that are necessary for effective emergency transport were presented to experts for agreement, 29 items were accepted for the final item set, 12 items revised, 17 new items added, and 26 items yielded a CVI of < 0.8 and thus eliminated, therefore there was 58 items remining for analysis in phase two.

***Round two.*** Fifty-eight items were presented to the experts to reflect on their ratings for those items that reached consensus in Delphi round one and asked them to re-rate the revised and new items. 56 items were accepted for the final item set, 4 items revised, one [1] new item added, and one [1] new item yielded a CVI of < 0.8 and thus eliminated, therefore final item set for a scale to measure components of effective emergency patient transport in rural health facilities consisted of 58 items.

### Phase three

All participants (*n* = 302) responded to all items of the RET instrument. In all the four districts, 308 were invited to participate, and 302 participated (98% response rate). Most participants (59.3%) were females, and most were aged between 31 and 40. All the nurses had their experience as a nurse the same as the transport nurse. Most (42.7%) participants worked in the clinics compared to the primary hospital and health post. Most (85.4%) of nurses were diploma holders compared to those with bachelor’s degrees, whereas most (72.5%) did not have a post-basic qualification, and 21.2% were midwives. **See** Table 3.

**Table 3:** *Descriptive data from Sample from instrument testing (* *n* *= 302)*

Variables	*n*	%
Gender		
Females	179	59.3
Males	123	40.7
Age		
< 30	90	29.8
31–40	116	38.4
41–50	67	22.2
51–60	23	7.6
> 60	6	2.0
Experience as a nurse		
< 5	83	32.5
6–10	80	28.5
11–15	52	16.2
16–20	35	10.6
> 20	52	12.3
Experience as a Transport Nurse		
< 5	98	32.5
6–10	86	28.5
11–15	49	16.2
16–20	32	10.6
> 20	37	12.3
**District/ Region**		
Boteti	75	24.8
Kgalagadi North	68	22.5
Kweneng West	81	26.8
Okavango	78	25.8
**Work Setting**		
Clinic	129	42.7
Health Post	80	26.5
Primary Hospital	93	30.8
**Qualification**		
Bachelors	44	14.6
Diploma	258	85.4
**Post Basic qualification**		
General nurses (None)	219	72.5
Midwifery	64	21.2
Psychiatry	8	2.6
Family Nurse Practitioner	11	3.6

**Fig. 2:**
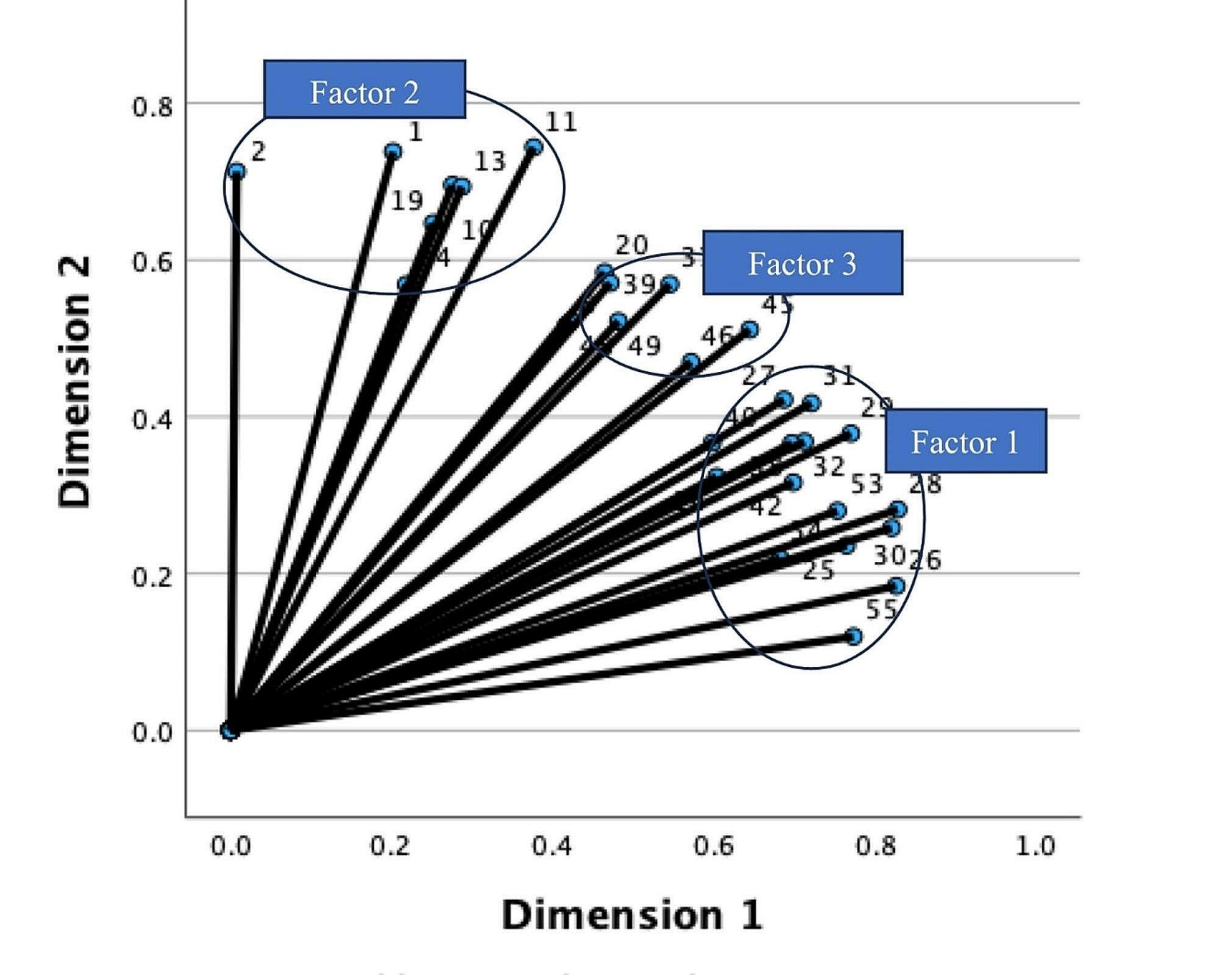
The variable map indicating three factors

The graphical representation of the factorial solution (Fig. 2) and the component loadings (Table 4) reveal three factors. Therefore, the categorical principal components analysis (CATPCA) method with Varimax rotation was performed to examine the factor structure of the three subscales of RET scale.

**Table 4:** *Rotated Factor Matrix Categorical Principal Component Analysis (CATPCA) of the 30-item RET scale (varimax rotation;* *N* *= 302)*

Factor 1: Knowledge & Skills: The ability to apply knowledge to specific situations by nurses working in rural clinics and health posts undertaking emergency patient transport. (9 items) Example of Items with Factor Loadings	
I am confident in my ability to handle emergencies in transport patients	**0.712**
My basic nursing degree/ diploma adequately prepared me to function in emergency care situations	**0.725**
I have enough people to help me during emergency patient transport.	**0.476**
I understand what emergency drugs are required for the transport of patients.	**0.693**
I am knowledgeable about most if not all essential emergency care equipment.	**0.717**
**Factor 2: Technology & Tools**: Objects used to assist persons in performing tasks. Tool and technology factors can be characteristics such as usability, accessibility (10 items) **Example of Items with Factor Loadings**	
My facility has access to vehicle/ transport in case of emergency.	**0.656**
Ambulances in my facility is always well equipped to transport emergency patient	**0.822**
There is a controlled checklist for an ambulance equipment.	**0.813**
There are sufficient emergency drugs to use during emergency patient transport.	**0.680**
The transport equipment is always in good condition to use during transfer.	**0.782**
**Factor 3: Organization**: The structure that provides and coordinates time, space, resources, and activities. (7 items) **Example of Items with Factor Loadings**	
There are standing order protocols for nurses involved in patient transport.	**0.524**
The Nurses Act addresses the expanded professional roles in emergency transport of patients.	**0.657**
There is an ambulance management system in the clusters and districts.	**0.658**
There is an adequate system to capture mortality data attributed to emergency transport.	**0.735**
There is a system to protect patient information.	**0.756**

© the full scale is available on request from the authors

***Factor Extraction of the RET Scale.*** A categorical principal components analysis factor analysis identified three components explaining 63.346% of the total variance. The first factor, technology, and tools, accounted for 27.316% of the total variance and included 14 items with factor loadings ranging from 0.535 to 0.822. The second factor, knowledge, and skills, had nine items with factor loadings ranging from 0.476 to 0.725, accounting for 18.15% of the total variance. The third factor (organization) explained 17.88% of the total variance and consisted of seven items with factor loadings of 0.524–0.756.

**Tests of Reliability.** The RET total Cronbach Alpha value was 0.980. The reliability of the three factors, as determined by the CATPCA’s default calculation of the Cronbach Alpha, was 0.960, 0.946, and 0.956, indicating that the items are highly correlated.

## Discussion

The study aimed to develop an instrument to measure and evaluate effective emergency patient transport. The recommendation of phase one of the study, the qualitative descriptive study, was that to ensure effective emergency transport, it is imperative to standardize emergency patient transports on a national level, built on consensus from emergency care experts [22].

The first dimension of **Tools and Technologies**, refers to objects used to assist persons in performing tasks and are characterized by usability, accessibility, availability, and portability. Importantly, our study findings showed that there should be access to an ambulance that is well-equipped to meet the requirements needed for safe task performance. Moreover, our study affirmed that a well-equipped ambulance should enable nurses to perform tasks such as having adequate lighting and a place to hang fluids. As much as the ambulance is a tool used to transport patients, it is also a place where tasks occur under specific organizational conditions such as layout, lighting, and workstation design [15]. The result of this study underscores the importance of a referral policy that is appropriate at all levels as well as well-coordinated emergency care. These findings concur with previous research emphasizing the need for an office to cover emergency care to coordinate services [23].

The study’s findings suggest that new nurses should receive assistance when transferring emergency cases to high-level facilities in remote regions. These results support earlier research conducted in Sweden, where single responder and assessment units have been implemented to meet the increasing need for ambulance assessment and care; single responders required a colleague when caring for critically ill patients, not only for practical reasons but also to share responsibility and to receive confirmation that their assessments were accurate [24]. Still in Sweden, through a qualitative study with 14 registered nurses, they reported that working with their competent colleagues, prehospital emergency nurses, reduced the feeling of loneliness and reduce stress [25]. The findings of this study also indicate that a communication system with the receiving facility before the transfer is essential to ensure a smooth transfer of care to healthcare providers at the receiving facility is essential. These findings concur with the qualitative descriptive study conducted in the United States of America, which aimed to characterize the experiences of inpatient floor-level bedside nurses caring for intrahospital (IHT) transfer patients and identify care coordination challenges and solutions with 21 nurses in an academic hospital. Nurses acknowledged that interhospital transfer started with a handoff report over the phone. As the first point of contact for patients at the time of arrival at the receiving facility, nurses expressed that, at times, it is difficult for nurses to understand why certain IHT patients were transferred [26]. However, they serve as the healthcare team spokesperson and are prepared to answer patient questions. As a result, this underscores the importance of effective communication for easy transition of care.

The second dimension is **Knowledge and Skills.** Our study findings show that knowledge and skills are necessary for effective emergency transport and that basic nursing training should adequately prepare transport nurses to function in emergency care situations. Similar to Indonesia, most nurses in Botswana are Diploma in nursing graduates, and supplementary content on emergency patient transport should also be introduced into the Diploma and Bachelor of Nursing curricula [27]. Continuous experience-based professional development, such as workshops, reflective peer discussions, and practical skills training, will further strengthen professional development to compensate for the nursing curriculum’s deficiencies [28].

The third dimension is **Organization.** These involved the management of health facilities and ambulance services, staffing of facilities and its effect on patient transport, collaboration between clusters and receiving facilities, management support, and teamwork. Importantly our study findings show that a system to capture mortality data attributed to emergency transport is important to provide an effective emergency patient transport. This will help to identify gaps and develop further management and prevention strategies [29].

### Strengths

This study selected four of the remotest, isolated health districts amongst the total of 27 health districts in Botswana, and this provided a more distinct understanding of what nurses regarded as important in providing effective emergency transport in rural areas of Botswana.

### Limitations

National experts assessed the content validity using modified Delphi techniques, although some experts had an international background, therefore generalizability of the research results could be limited. The result of four districts out of 27 health districts does not claim generalizability, however this study strived to use four remotest districts to increase transferability to contexts with similar characteristics. Confirmatory factor analysis was not undertaken due to the limited level of data, to be split into two subscales. However, the sample was acceptable for psychometric evaluation; therefore, the validity is only satisfactory. Moreover, test-retest reliability could not be assessed due to a cross-sectional study design. Therefore, some aspects of validity and reliability were not addressed in the present study, rendering a further evaluation of the psychometric properties of the RET scale.

### Implications for rural emergency transport

The study highlights the importance of exploring the necessities for effective emergency patient transport and standardizing it nationally, based on consensus from emergency care experts. The Scale for Measuring Effective Rural Emergency Transfer (RET) can be used as an outcome measure in assessing the effectiveness of emergency patient transport. Moreover, it can help identify the gaps in the components of the work system and their influence on the care process during transportation.

The RET scale helps develop programs to educate rural emergency transport providers. It can also guide rural healthcare providers to gain the knowledge and competencies they need to provide effective emergency patient transport.

## Conclusion

The current study proposes a self-report scale that considers technology and equipment, knowledge and skills, and organization to evaluate the efficiency of rural emergency transport. The results show that the RET Safety Scale has acceptable initial psychometric features among a sample of rural nurses, even if more validation research on the scale is required to establish its psychometric properties across various districts in Botswana. This instrument can be utilized to improve clinical practice and gain a better understanding of emergency transport in rural healthcare facilities.

### Electronic supplementary material

Below is the link to the electronic supplementary material.

Supplementary Material 1

Supplementary Material 2

## Data Availability

The data that support the findings of this study are available from the corresponding author on reasonable request.
