# Supplementary material for: Development and psychometric testing of a scale to measure effective rural emergency transfer (RET)
Source: BMC Emerg Med. 2024 Jul 29;24:131. doi: 10.1186/s12873-024-01046-2 (PMC11287921; doi:10.1186/s12873-024-01046-2)
Supplement: Supplementary file 1 — Supplementary Material 1 [file 12873_2024_1046_MOESM1_ESM.docx]

**Development and Initial Psychometric Testing of a Scale Measuring Effective Emergency Patient Transport in Rural Health Facilities.**

You are being invited to participate in a Delphi study. The aim of the study is to ascertain consensus regarding the knowledge and skills required for nurses to provide safe emergency patient transport in Botswana by looking at the viewpoints of experts in the field of emergency care. We have recognized you as an expert in your field and are inviting you to join our core group and take part in this study. We would value your expert opinion.

**What you should know about this research study:**

- We give you this informed consent document so that you may read about the purpose, risks, and benefits of this research study.
- You have the right to refuse to take part or agree to take part now and change your mind later.
- Please review this consent form carefully. Ask any questions before you decide.
- Your participation is voluntary.

**RISKS AND DISCOMFORTS**

Participation involves no risks to you.

**BENEFITS AND/OR COMPENSATION**

There is no cost for the participants. You will not receive direct benefits from participating in this study. This study will help develop a more distinct understanding of nurses' experiences with emergency transport in rural areas of Botswana. It can serve as the realistic baseline for creating teaching and training materials that address the population's complex needs and setting.

**CONFIDENTIALITY**

The data from this investigation will be kept private and confidential. Individual information will not be shared with anyone outside the research study group. We will not collect names, contact information, or Omang numbers. None of these will be used for commercial use.

**VOLUNTARY PARTICIPATION**

Participation is voluntary. You can withdraw from participating at any time while completing the survey by closing your browser; however, once the completed survey has been submitted, it will not be possible to withdraw your information. Any information recorded before you withdraw will be used by the researchers for the purposes of the study, but no information will be collected after you withdraw your permission.

**Questions**: The investigator or a member of the research team will try to answer all of your questions. If you have questions or concerns at any time, contact Ms. Tebogo Mamalelala at +267 74649810 or Dr. Holzemer at holzemer@sn.rutgers.edu. Contact Tebogo Mamalelala at [ttm37@sn.rutgers.edu](mailto:ttm37@sn.rutgers.edu) if there is no answer at that phone number.

If you have any questions concerning this study or consent form beyond those answered by the investigator, including questions about the research, your rights as a research participant; or if you feel that you have been treated unfairly and would like to talk to someone other than a member of the research team, please feel free to contact the Office of Research and Development, University of Botswana, Phone: Ms Dimpho Njadingwe on 355-2900, E-mail: ORD@ub.ac.bw, Telefax: [0267] 395-7573.

**By proceeding with this survey, you indicate your consent to participate in the study as described.**

**Development and Initial Psychometric Testing of a Scale Measuring Effective Emergency Patient Transport Rural Health Facilities.**

The conceptual model for the scale measuring safe emergency patient transport was based on the SEIPS model of the work system and patient safety (SEIPS). The (SEIPS) model builds on the Donabedian model expanding the structure component to include aspects of the work system and patient, employee, and organizational outcomes. The model is practice-driven and asserts that work systems are multidimensional and comprise five distinct dimensions: person, technology and tools, tasks, organization, and physical environment (Carayon et al., 2006). The items were generated and synthesized from an extensive literature review and from the qualitative descriptive study. The descriptive qualitative technique employing interviews analyzed the first-hand experiences of nurses in rural emergency patient transportation. The interviews explored the range of skills and knowledge nurses use on the job, the complex needs of this demographic, the resources they need, and the problems they encounter in certain situations. The table below defines the dimensions:

**Table 1**: Dimensions and their definitions

| Dimension | Definitions |
| --- | --- |
| Person | The nurse working in rural clinics and health posts undertaking emergency patient transport. The person component of the work system structure includes factors such as education, skills, knowledge, communication, motivation, and physical and psychological characteristics. |
| Technology and tools | Objects used to assist persons in performing tasks. Tool and technology factors in the SEIPS model can be characteristics such as usability, accessibility availability, and portability. |
| Organisation | The structure that provides and coordinates time, space, resources and activities. |
| Tasks | Tasks performed before transport, during transport, and on arrival at the receiving facility, this includes task complexity, difficulty, ambiguity and sequence. |
| Environment | Physical work setting where tasks are performed that influences the care process. |
| Patient outcomes: | Patient safety, and quality of Care |
| Employee and Organizational outcomes | Job satisfaction and other attitudes, job stress and burnout, employee safety and health, and turnover. |

**INSTRUCTIONS**

The aim of the study is to ascertain consensus regarding the knowledge and skills required for nurses to provide safe emergency patient transport in Botswana by examining the viewpoints of experts in the field of emergency care. We have recognized you as an expert in your field and are inviting you to join our Core group and take part in this study. We would value your expert opinion.

The instrument is designed to measure emergency patient transport safety by nurses in rural health facilities. there are two subsequent online questionnaires for you to complete. The second survey will be a revised version of the initial survey, based upon the core experts’ opinions and input. There will be a 3-week gap between each of the two online surveys. The link to the survey will go inactive after three weeks. Email reminders will be issued 7 and 14 days after the survey has been administered.

In the first round, items from the qualitative descriptive study and literature review were generated and categorized into seven dimensions following the SIEPS model. You are asked to reflect your degree of agreement with the statement on a five-point Likert scale (1 being ‘never,’ 2 ‘rarely’, 3 ‘sometimes,’ 4 ‘often,’ and 5 ‘always’). You will also be asked for comments regarding the wording and clarity and suggestions for new criteria that you think should be added. In round two, you will be given an updated version of the list from round One and asked to reflect your degree of agreement with the statement on a 5-point scale from 1 (Never) to 5 (Always) for each item. In addition to the rating scales, you can add additional free text comments to help refine criterion and answer options.

**Demographics**

Do you agree to participate in this study?

- Yes, I agree to participate in this study.
- No, I do not wish to participate in this study.

What is your professional designation?

- Emergency nurse
- Paramedic
- Emergency doctor
- Surgeon
- Other please specify…….

What is your primary site of work?

- Teaching facility/ Academic setting
- Clinical setting / Practice
- Both

Highest level academic of qualification

- Bachelor’s degree
- Master’s degree
- PhD

What is your age?

- Below 30
- 31-40
- 41-50
- 51-60
- Above 60

What is your gender?

- Female
- Male
- Prefer not to say

| **Domain (s)** | **Items** | Please circle a number from 1 (Never) to 5 (Always) for each item reflecting your **DEGREE of AGREEMENT** with the statement | | | | | **Comments/ Suggested revision** |
| --- | --- | --- | --- | --- | --- | --- | --- |
| **Person/**  **Transporter** |  | **Never**  **1** | **Rarely**  **2** | **Sometimes**  **3** | **Often**  **4** | **Always**  **5** |  |
|  | 1. I am confident in my ability to handle challenging emergencies. | **1** | **2** | **3** | **4** | **5** |  |
|  | 1. My foundational nursing degree/ diploma adequately prepared me to function in   emergency care. | **1** | **2** | **3** | **4** | **5** |  |
|  | 1. I can identify a patient who needs emergency patient transfer. | **1** | **2** | **3** | **4** | **5** |  |
|  | 1. I initiate emergency patient transfer | **1** | **2** | **3** | **4** | **5** |  |
|  | 1. There is a colleague to assist with a challenging case whenever I am on call. | **1** | **2** | **3** | **4** | **5** |  |
|  | 1. I have sufficient manpower to assist during emergency patient transport. | **1** | **2** | **3** | **4** | **5** |  |
|  | 1. I advocate for my patients at the receiving facility. | **1** | **2** | **3** | **4** | **5** |  |
|  | 1. I need continuous education in emergency care. | **1** | **2** | **3** | **4** | **5** |  |
|  | 1. I understand how and when to use emergency drugs. | **1** | **2** | **3** | **4** | **5** |  |
|  |  | **Never** | **Rarely** | **Sometimes** | **Often** | **Always** |  |
|  | 1. I am knowledgeable about using medical equipment. | **1** | **2** | **3** | **4** | **5** |  |
|  | 1. I need continuous training in labor and delivery. | **1** | **2** | **3** | **4** | **5** |  |
|  | 1. I need training in Emergency Obstetric and New-born Care (EmONC). | **1** | **2** | **3** | **4** | **5** |  |
|  | 1. There is an easy-care transition to the nurses in the receiving facilities | **1** | **2** | **3** | **4** | **5** |  |
|  | 1. There is a clear standardized handover procedure | **1** | **2** | **3** | **4** | **5** |  |
|  | 1. I have CPR and heart attack/cardiac arrest management skills | **1** | **2** | **3** | **4** | **5** |  |
|  | 1. I need to be trained in Basic Life Support (BLS) | **1** | **2** | **3** | **4** | **5** |  |
|  | 1. There is need to train support staff such as drivers, cleaners and nightwatchmen in first aid. | **1** | **2** | **3** | **4** | **5** |  |
|  | 1. I need ongoing emotional and psychosocial support and counseling. | **1** | **2** | **3** | **4** | **5** |  |
| **Tasks and**  **Conditions** | 1. I initiate management of time sensitive conditions such as myocardial infarction and sepsis | **1** | **2** | **3** | **4** | **5** |  |
|  | 1. I perform lifesaving procedures in patients with drug overdose such as gastric lavage. | **1** | **2** | **3** | **4** | **5** |  |
|  |  | **Never** | **Rarely** | **Sometimes** | **Often** | **Always** |  |
|  | 1. I safely provide lifesaving treatment using Basic Life Support (BLS) | **1** | **2** | **3** | **4** | **5** |  |
|  | 1. I initiate antidotes for patients who overdosed drugs. | **1** | **2** | **3** | **4** | **5** |  |
|  | 1. I manage labor and delivery conditions | **1** | **2** | **3** | **4** | **5** |  |
|  | 1. I manage pre-partum and post-partum bleeding management | **1** | **2** | **3** | **4** | **5** |  |
|  | 1. I manage preeclampsia and eclampsia | **1** | **2** | **3** | **4** | **5** |  |
|  | 1. I resuscitate the infant | **1** | **2** | **3** | **4** | **5** |  |
|  | 1. I manage abortion related complications | **1** | **2** | **3** | **4** | **5** |  |
|  | 1. I control bleeding in trauma cases | **1** | **2** | **3** | **4** | **5** |  |
|  | 1. I immobilize long bone fractures | **1** | **2** | **3** | **4** | **5** |  |
|  | 1. I immobilize cervical spine | **1** | **2** | **3** | **4** | **5** |  |
|  | 1. I manage of poisoning/ ingestions e.g., organophosphate poisoning. | **1** | **2** | **3** | **4** | **5** |  |
|  | 1. I manage drug overdose e.g., paracetamol overdose. | **1** | **2** | **3** | **4** | **5** |  |
|  | 1. I manage envenomation (Snake Bite and Scorpion Bite) | **1** | **2** | **3** | **4** | **5** |  |
|  | 1. I initiate appropriate fluid resuscitation for burn patients | **1** | **2** | **3** | **4** | **5** |  |
|  |  | **Never** | **Rarely** | **Sometimes** | **Often** | **Always** |  |
|  | 1. I manage dehydration in children | **1** | **2** | **3** | **4** | **5** |  |
|  | 1. I manage complicated malaria | **1** | **2** | **3** | **4** | **5** |  |
|  | 1. I manage seizures/ convulsions | **1** | **2** | **3** | **4** | **5** |  |
|  | 1. I administer oxygen | **1** | **2** | **3** | **4** | **5** |  |
|  | 1. I manage diabetic ketoacidosis | **1** | **2** | **3** | **4** | **5** |  |
|  | 1. I manage psychiatric emergencies | **1** | **2** | **3** | **4** | **5** |  |
|  | 1. I manage heart attack/cardiac arrest | **1** | **2** | **3** | **4** | **5** |  |
|  | 1. I transport dead bodies for declaration at a higher facility. | **1** | **2** | **3** | **4** | **5** |  |
| **Technology and Tools** | 1. The facility has access to well-equipped ambulance in case of emergency | **1** | **2** | **3** | **4** | **5** |  |
|  | 1. The facility has access to vehicle/ transport in case of emergency | **1** | **2** | **3** | **4** | **5** |  |
|  | 1. The ambulance is available on time in case of emergency. | **1** | **2** | **3** | **4** | **5** |  |
|  | 1. The ambulance used to transport patients is suitable has a stretchers/bed. | **1** | **2** | **3** | **4** | **5** |  |
|  | 1. The ambulance used to transport patients has a designated area for oxygen cylinder | **1** | **2** | **3** | **4** | **5** |  |
|  | 1. The ambulance used to transport patient has a place to hang IV fluids. | **1** | **2** | **3** | **4** | **5** |  |
|  | 1. There are sufficient emergency drugs to use during emergency patient transport | **1** | **2** | **3** | **4** | **5** |  |
|  |  | **Never** | **Rarely** | **Sometimes** | **Often** | **Always** |  |
|  | 1. There is adequate medical equipment to use during emergency patient transport | **1** | **2** | **3** | **4** | **5** |  |
|  | 1. I am able to monitor the vital signs during patient transport. | **1** | **2** | **3** | **4** | **5** |  |
|  | 1. The transport equipment is reliable | **1** | **2** | **3** | **4** | **5** |  |
|  | 1. The transport equipment meets the requirements needed to perform the transport safely. | **1** | **2** | **3** | **4** | **5** |  |
|  | 1. There is always an oxygen cylinders to use in case of emergency | **1** | **2** | **3** | **4** | **5** |  |
|  | 1. There is sufficient personal protective equipment to care for high-risk patients during emergency patient transport. | **1** | **2** | **3** | **4** | **5** |  |
|  | 1. It is easy to stabilise the patient and monitor the patient during emergency patient transport. | **1** | **2** | **3** | **4** | **5** |  |
|  | 1. I always have basic diagnostic materials and equipment such as pregnancy, COVID, HIV and malaria test kit for evaluation before transfer | **1** | **2** | **3** | **4** | **5** |  |
| **Organization** | 1. There is a referral policy that is appropriate for all settings. | **1** | **2** | **3** | **4** | **5** |  |
|  | 1. There is a system that coordinates emergency care at the clinics and health posts. | **1** | **2** | **3** | **4** | **5** |  |
|  |  | **Never** | **Rarely** | **Sometimes** | **Often** | **Always** |  |
|  | 1. There is a means of communication with the receiving facility before transfer. | **1** | **2** | **3** | **4** | **5** |  |
|  | 1. There is an organized triage system that does not rely on the decision to transfer being based on nurses’ discretion | **1** | **2** | **3** | **4** | **5** |  |
|  | 1. An organized reliable advisory service regarding patient initial management and clinical support prior transfer is available | **1** | **2** | **3** | **4** | **5** |  |
|  | 1. There are standing order protocols for nurses involved in patient transport. | **1** | **2** | **3** | **4** | **5** |  |
|  | 1. Ongoing continued training to develop new knowledge and prepare nurses to provide quality and safe emergency care during patient transport are available | **1** | **2** | **3** | **4** | **5** |  |
|  | 1. Nurses take on expanded professional roles in emergency care. | **1** | **2** | **3** | **4** | **5** |  |
|  | 1. Nurses have the capacity to adequately address patients needing emergency care | **1** | **2** | **3** | **4** | **5** |  |
|  | 1. There is adequate nurse staffing, in the clinics and health posts | **1** | **2** | **3** | **4** | **5** |  |
|  | 1. The system allows to declare and pronounce death | **1** | **2** | **3** | **4** | **5** |  |
|  | 1. There is induction and orientation of nurses to remote areas. | **1** | **2** | **3** | **4** | **5** |  |
|  | 1. There is support for new nurses assigned to remote areas | **1** | **2** | **3** | **4** | **5** |  |
|  | 1. There is ambulance management system in the clusters and districts | **1** | **2** | **3** | **4** | **5** |  |
| **Internal Environment/Ambulance Layout/**  **External Environment** | 1. The ambulance enables the nurse to provide privacy during transport. | **1** | **2** | **3** | **4** | **5** |  |
|  | 1. The ambulance enables the nurse to provide privacy during transport. | **1** | **2** | **3** | **4** | **5** |  |
|  | 1. There is adequate lighting in the ambulance during emergency patient transport | **1** | **2** | **3** | **4** | **5** |  |
|  | 1. There is consistent lighting and electricity at the clinics to facilitate stabilization of patient before patient transfer. | **1** | **2** | **3** | **4** | **5** |  |
|  | 1. There is access to phones to use to communicate with receiving facilities before patient transfer | **1** | **2** | **3** | **4** | **5** |  |
|  | 1. Ambulances are equipped to manage the challenging terrain in the rural areas. | **1** | **2** | **3** | **4** | **5** |  |
|  | 1. There are reliable mobile networks in the rural clinics and health posts | **1** | **2** | **3** | **4** | **5** |  |
| **Patient Outcomes** | 1. There is timely transfer of emergency patients | **1** | **2** | **3** | **4** | **5** |  |
|  | 1. The patients and family members are satisfied with patient transfer. | **1** | **2** | **3** | **4** | **5** |  |
|  |  | **Never** | **Rarely** | **Sometimes** | **Often** | **Always** |  |
|  | 1. Patients are comfortable during patient transport. | **1** | **2** | **3** | **4** | **5** |  |
|  | 1. There is an adequate system to capture mortality data attributed to emergency transport. | **1** | **2** | **3** | **4** | **5** |  |
| **Employee And Organizational Outcomes** | 1. The system supports referral process | **1** | **2** | **3** | **4** | **5** |  |
|  | 1. Nurses who work in rural health facilities are satisfied with their working conditions. | **1** | **2** | **3** | **4** | **5** |  |
