# Supplementary material for: Development and psychometric testing of a scale to measure effective rural emergency transfer (RET)
Source: BMC Emerg Med. 2024 Jul 29;24:131. doi: 10.1186/s12873-024-01046-2 (PMC11287921; doi:10.1186/s12873-024-01046-2)
Supplement: Supplementary file 2 — Supplementary Material 2 [file 12873_2024_1046_MOESM2_ESM.docx]

**Development and Initial Psychometric Testing of a Scale Measuring Effective Emergency Patient Transport from Rural Health Facilities.**

**INSTRUCTIONS**

My name is Tebogo Mamalelala, a PhD candidate at Rutgers School of Nursing. You are invited to take part in a research study. This study was approved by University of Botswana, Ministry of Health and Rutgers University. The study will inform the development and evaluation of psychometric properties of a scale measuring effective emergency patient transport from rural health facilities. You are asked to reflect on your own experience with emergency patient transport and highlight the significance of each statement or item. The bipolar Likert scale with descriptors indicates five levels of agreement (strongly disagree to strongly agree). Higher scores indicate more significant agreement that the item is essential to provide effective emergency patient transport.
If you have any questions about this study or your rights as a participant, either while participating or after you have completed the study, you should contact:

Tebogo Mamalelala: 74649810

**Please select one response for each demographic item**.

**Demographics**

What is your work setting?

- Clinic
- Health post

In which health district is your health facility located

- Kweneng West
- Kgalagadi North
- Okavango
- Boteti

How long have you been in the current facility?

- 0-5
- 6-10
- 11-15
- 16-20
- > 20

What is your gender?

- Female
- Male
- Prefer not to say

What are your years of experience as a nurse?

- 0-5
- 6-10
- 11-15
- 16-20
- > 20

What are your years of experience as an escorting nurse?

- 0-5
- 6-10
- 11-15
- 16-20
- > 20

What is your age?

- Below 30
- 31-40
- 41-50
- 51-60
- Above 60

Highest level academic of qualification?

- Diploma
- Bachelor’s degree
- Master’s degree

What is your post basic Qualification?

- Midwifery
- Psychiatry
- Family nurse practitioner
- Community health nurse
- None
- Other; specify………………...

Please circle one number for each item listed that expresses your **Degree of Agreement**

| Items | Strongly Disagree | Disagree | Neutral | Agree | Strongly Agree |
| --- | --- | --- | --- | --- | --- |
| Scale | 1 | 2 | 3 | 4 | 5 |
| 1. I am confident in my ability to handle emergencies in transport patients | 1 | 2 | 3 | 4 | 5 |
| 1. My basic nursing degree/ diploma adequately prepared me to function in emergency care situations | 1 | 2 | 3 | 4 | 5 |
| 1. I have formal training in safe transport of emergency patients | 1 | 2 | 3 | 4 | 5 |
| 1. I practice within my scope during patient transport. | 1 | 2 | 3 | 4 | 5 |
| 1. I have the knowledge and skill to manage basic emergencies which may arise during transportation. e.g being able to check for hypoglycemia and manage it. | 1 | 2 | 3 | 4 | 5 |
| 1. I can identify patients who require emergency transport | 1 | 2 | 3 | 4 | 5 |
| 1. There is a colleague in my facility to assist with a challenging case whenever I am on call | 1 | 2 | 3 | 4 | 5 |
| 1. I have enough people to help me during emergency patient transport. | 1 | 2 | 3 | 4 | 5 |
| 1. Nurses need continuous education in the emergency transport of patients. | 1 | 2 | 3 | 4 | 5 |
| 1. I understand what emergency drugs are required for the transport of patients. | 1 | 2 | 3 | 4 | 5 |
| 1. I am knowledgeable about most if not all essential emergency care equipment. | 1 | 2 | 3 | 4 | 5 |
| 1. There is a clear standardized handover procedure. | 1 | 2 | 3 | 4 | 5 |
| 1. I have CPR and cardiac arrest management skills. | 1 | 2 | 3 | 4 | 5 |
| 1. There is a need to train support staff such as health care auxiliaries, drivers, cleaners, and nightwatchmen in first aid. | 1 | 2 | 3 | 4 | 5 |
| 1. I can cope with stressful situations during emergency patient transport. | 1 | 2 | 3 | 4 | 5 |
| 1. The ambulance allows proper and safe disposal of sharps and waste. | 1 | 2 | 3 | 4 | 5 |
| 1. I perform tasks during emergency patient transport. e.g., conducting deliveries | 1 | 2 | 3 | 4 | 5 |
| 1. I always monitor the vital signs during patient transport. | 1 | 2 | 3 | 4 | 5 |
| 1. I can monitor patient's clinical condition during emergency patient transport. | 1 | 2 | 3 | 4 | 5 |
|  | Strongly Disagree | Disagree | Neutral | Agree | Strongly Agree |
| 1. Transfer of care to health care providers at the receiving facility is easily accomplished. | 1 | 2 | 3 | 4 | 5 |
| 1. I advocate for my patients at the receiving facility. | 1 | 2 | 3 | 4 | 5 |
| 1. I handle competing tasks while transporting emergency patients. | 1 | 2 | 3 | 4 | 5 |
| 1. I manage time pressure during emergency patient transport. | 1 | 2 | 3 | 4 | 5 |
| 1. My facility has access to vehicle/ transport in case of emergency. | 1 | 2 | 3 | 4 | 5 |
| 1. Ambulances in my facility is always well equipped to transport emergency patient | 1 | 2 | 3 | 4 | 5 |
| 1. There is a controlled checklist for an ambulance equipment. | 1 | 2 | 3 | 4 | 5 |
| 1. There are sufficient emergency drugs to use during emergency patient transport. | 1 | 2 | 3 | 4 | 5 |
| 1. The transport equipment is always in good condition to use during transfer. | 1 | 2 | 3 | 4 | 5 |
| 1. I have basic diagnostic materials and equipment available to me in a transfer. | 1 | 2 | 3 | 4 | 5 |
| 1. There is sufficient personal protective equipment to care for high-risk patients during emergency patient transport. | 1 | 2 | 3 | 4 | 5 |
| 1. There is a patient referral policy that is appropriate for all settings. | 1 | 2 | 3 | 4 | 5 |
| 1. There is a system that coordinates emergency care at the clinics and health posts. | 1 | 2 | 3 | 4 | 5 |
| 1. The health system supports referring patients to higher levels of care without following the ladder of referral when necessary. | 1 | 2 | 3 | 4 | 5 |
| 1. There is a means of communication with the receiving facility before transfer. | 1 | 2 | 3 | 4 | 5 |
| 1. There is a triage system for transfer that does not rely on the decision to transfer being based on nurses’ discretion. | 1 | 2 | 3 | 4 | 5 |
| 1. An organized reliable advisory service regarding patient initial management and clinical support before transfer is available. | 1 | 2 | 3 | 4 | 5 |
| 1. There are standing order protocols for nurses involved in patient transport. | 1 | 2 | 3 | 4 | 5 |
|  | Strongly Disagree | Disagree | Neutral | Agree | Strongly Agree |
| 1. Ongoing continued training to develop new knowledge and prepare nurses to provide quality and safe emergency care during patient transport is available. | 1 | 2 | 3 | 4 | 5 |
| 1. The Nurses Act addresses the expanded professional roles in emergency transport of patients. | 1 | 2 | 3 | 4 | 5 |
| 1. There is support for new nurses in remote areas during the transfer of emergency cases to high level facilities. | 1 | 2 | 3 | 4 | 5 |
| 1. Nurses have the capacity to adequately address patients needing emergency care during transport. | 1 | 2 | 3 | 4 | 5 |
| 1. There is adequate nursing staff in clinics/health posts to allow safe patient transportation and still allow facility coverage. | 1 | 2 | 3 | 4 | 5 |
| 1. The system allows nurses to declare and pronounce death. | 1 | 2 | 3 | 4 | 5 |
| 1. There is induction and orientation of nurses to remote areas. | 1 | 2 | 3 | 4 | 5 |
| 1. There is an ambulance management system in the clusters and districts. | 1 | 2 | 3 | 4 | 5 |
| 1. There is an adequate system to capture mortality data attributed to emergency transport. | 1 | 2 | 3 | 4 | 5 |
| 1. Medical items in the ambulance are based on the guidelines. | 1 | 2 | 3 | 4 | 5 |
| 1. There is a system to protect patient information. | 1 | 2 | 3 | 4 | 5 |
| 1. There is a system to identify patients who are being transported e.g., name tags. Identification badge | 1 | 2 | 3 | 4 | 5 |
| 1. There is a policy addressing the safety of the nurses involved in emergency patient transport including insurance and compensation. | 1 | 2 | 3 | 4 | 5 |
| 1. The ambulance enables the nurse to provide privacy during transport. | 1 | 2 | 3 | 4 | 5 |
| 1. The available ambulances can navigate difficult terrain. | 1 | 2 | 3 | 4 | 5 |
| 1. There is adequate lighting in the ambulance during emergency patient transport. | 1 | 2 | 3 | 4 | 5 |
|  | Strongly Disagree | Disagree | Neutral | Agree | Strongly Agree |
| 1. The ambulance used to transport patients has a designated area for an oxygen cylinder. | 1 | 2 | 3 | 4 | 5 |
| 1. The ambulance used to transport patients has a place to hang IV fluids. | 1 | 2 | 3 | 4 | 5 |
| 1. There is consistent lighting and electricity at the clinics to facilitate the stabilization of patient before patients transfer. | 1 | 2 | 3 | 4 | 5 |
| 1. There is access to phones to use to communicate with receiving facilities before patient transfer. | 1 | 2 | 3 | 4 | 5 |
| 1. There are reliable mobile networks in my health facility. | 1 | 2 | 3 | 4 | 5 |

**Thank you very much for participation!**

**
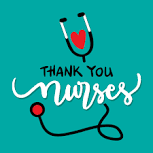
**
